# Large-scale transcriptome sequencing and gene analyses in the crab-eating macaque (*Macaca fascicularis*) for biomedical research

**DOI:** 10.1186/1471-2164-13-163

**Published:** 2012-05-04

**Authors:** Jae-Won Huh, Young-Hyun Kim, Sang-Je Park, Dae-Soo Kim, Sang-Rae Lee, Kyoung-Min Kim, Kang-Jin Jeong, Ji-Su Kim, Bong-Seok Song, Bo-Woong Sim, Sun-Uk Kim, Sang-Hyun Kim, Kyu-Tae Chang

**Affiliations:** 1 National Primate Research Center, Korea Research Institute of Bioscience and Biotechnology (KRIBB), Ochang, Chungbuk, 363-883, Republic of Korea; 2 Department of Biological Sciences, College of Natural Sciences, Pusan National University, Busan, 609-735, Republic of Korea; 3 University of Science & Technology, National Primate Research Center, KRIBB, Daejeon, 305-806, Republic of Korea; 4 Genome Resource Center, Korea Research Institute of Bioscience and Biotechnology (KRIBB), Daejeon, 305-806, Republic of Korea

## Abstract

**Background:**

As a human replacement, the crab-eating macaque (*Macaca fascicularis*) is an invaluable non-human primate model for biomedical research, but the lack of genetic information on this primate has represented a significant obstacle for its broader use.

**Results:**

Here, we sequenced the transcriptome of 16 tissues originated from two individuals of crab-eating macaque (male and female), and identified genes to resolve the main obstacles for understanding the biological response of the crab-eating macaque. From 4 million reads with 1.4 billion base sequences, 31,786 isotigs containing genes similar to those of humans, 12,672 novel isotigs, and 348,160 singletons were identified using the GS FLX sequencing method. Approximately 86% of human genes were represented among the genes sequenced in this study. Additionally, 175 tissue-specific transcripts were identified, 81 of which were experimentally validated. In total, 4,314 alternative splicing (AS) events were identified and analyzed. Intriguingly, 10.4% of AS events were associated with transposable element (TE) insertions. Finally, investigation of TE exonization events and evolutionary analysis were conducted, revealing interesting phenomena of human-specific amplified trends in TE exonization events.

**Conclusions:**

This report represents the first large-scale transcriptome sequencing and genetic analyses of M. fascicularis and could contribute to its utility for biomedical research and basic biology.

## Background

Crab-eating macaques (*Macaca fascicularis*) are one of the most frequently used and studied species for biomedical research [1]. Due to the broad range of habitats, they have various common names including crab-eating macaque, cynomolgus macaque, Philippine monkey, and long-tailed macaque. Numerous wild crab-eating macaques are distributed in Southeast Asia, including Indonesia, Philippines, Myanmar, Vietnam, and Thailand [2]. They inhabit various habitats including primary, secondary, coastal, mangrove, and riverine forests and areas near villages. Diurnal and arboreal crab-eating macaques belong to the infraorder *Catarrhini*, superfamily *Carecopithecoidea*, family *Cercopithecidae*, and genus *Macaca*.

With the aid of fossil records and comparative DNA sequence analysis, genus macaques and humans have diverged from a common ancestor between 25 and 31 million years ago [3]. This evolutionary relationship has made this primate as a more suitable experimental animal model than rodents, dogs, and pigs and may lead to its widespread use for the translational studies for drug testing [1]. Among the genus *Macaca,* Rhesus and crab-eating macaque is representative species which were widely used as a non-human primate model for biomedical research. However, the rhesus macaque is the most frequently used primate as a non-human primate model [4]. In the United States, more than 60% of monkeys housed in National Institutes of Health (NIH)-supported facilities are rhesus macaques [5]. Furthermore, 65% of the monkeys used for experimental research each year are rhesus macaques. In 2007, first draft genome sequences of rhesus macaque genome was published [4]. These worldwide trends in use and accumulated genome information data may lead to the assumption that the rhesus macaque is the ideal non-human primate model. However, the event of “export ban of rhesus monkey from India in 1977” had restricted the usage of Indian subspecies of the rhesus macaque and accelerate the building of self-sustaining breeding colonies in the US. Therefore, researchers who want to have a research with rhesus monkey in the outside of US have some problems, they have concerned the chinese-origin rhesus macaque and crab-eating macaque from south asia [6]. Furthermore, the crab-eating macaque has important advantages, including (1) easy handling derived from a smaller body size (♂ 412–648 mm, ♀ 385–503 mm vs. ♂ 483–635 mm, ♀ 470–531 mm), weight (♂ 4.7–8.3 kg, ♀ 2.5–5.7 kg vs. ♂ 5.6–10.9 kg, ♀ 4.4–10.9 kg) and longer tails than rhesus macaques [7]; (2) low cost and easy availability for experimental use; and (3) lack of seasonal fertility, which may affect efficient experiments and scheduling in the large-scale housing of experimental monkeys [8]. Finally, abundant gene information is available for the crab-eating macaque. Greater numbers of EST and full-length cDNA library sequences are available in the NCBI database for crab-eating macaque [9-15]. And recently their draft genome sequences also available in the EBI database [6,16]. Therefore, crab-eating macaque could be a excellent experimental primate animal models for biomedical studies.

In an in-depth examination of the published papers from 2010 to 2011 indicated that pharmacology field for safety and toxicity testing of newly developed drugs was the most frequently encountered [17-20]. In particular, the crab-eating macaque was used predominantly in brain research, the neurosciences, and clinical research [21-24]. Furthermore, experimental primate model have been developed by four different ways of simple replacement, induced, infection, and surgical. The induced method involved treatment with specific chemicals (e.g., 1-methyl-4-phenyl-1,2,3,6-tetrahydropyridine (MPTP) or streptozotocin (STZ)[25-27], whereas the surgical method (e.g., middle cerebral artery occlusion model for ischemia) were created through specific types of surgery [28]. The infection method was simpler than previously described since humans and the crab-eating macaque have numerous “anthroponosis” (the opposite of “zoonosis”), including influenza, tuberculosis, and hepatitis [29]. Lastly, simple replacement method was the usage of natural crab-eating monkey for specific purpose (e.g., drug safety or efficacy testing) [30].

From now, numerous disease models, including aging, alcohol abuse, Alzheimer’s disease, amenorrhea, asthma, diabetes, epilepsy, menopause, obesity, osteoporosis, Parkinson’s disease, plague, variola, vascular disease, and various infection disease models, have been developed and used [31-46] However, small amount of transcript sequences of crab-eating macaque could be a weak point to be a good experimental animals for biomedical application. If we have abundant transcript sequences for crab-eating macaque, we could design the whole gene probe sequences for microarray analyses. And also, due to the insufficient transcript sequences, we could not analyze the alternatively spliced transcripts in different tissues. Recent accumulated transcriptome information underlined that AS event is an important molecular mechanism since it can generate different functional units for transcriptome and proteome diversity using limited genetic sources[47-49]. And also human transcriptome studies with different human tissues show different AS patterns derived by tissue-specific alternative promoters and polyadenylation [50-52]. However, sometimes aberrant changes in alternative splicing could occur the human disease (e.g. retinitis pigmentosa or cystic fibrosis) [53,54]. And A few number of papers have reviewed the association between alternative splicing and disease [55-58]. Among the different AS mechanism, TE exonization is intriguing AS events [59]. Specifically, small amount of TEs show the tissue specific and species specific characters [60]. That means that TE exonization event could be a one of the important AS events. Therefore, AS is not a simple molecular aspect of RNA transcription, rather it represents a highly controlled and evolved molecular mechanism for generating genetic diversity using limited DNA resources. And also AS control mechanism are major growing topics in biomedical researches. Hence, the investigation of the AS events in specific genes is another means of novel gene or disease gene identification and characterization steps. However, these kinds of applications with crab-eating macaque for advanced biomedical research could be achieved by the massive amount of transcript sequences and information.

In this study, we carried out a whole-transcriptome sequencing analysis of 16 tissues from *Macaca fascicularis* using GS FLX sequencing to generate massive transcript information for the improvement of biomedical use. More than 4 million raw reads were created and assembled, resulting in 35,524 isogroups, 44,458 isotigs, 54,858 contigs, and 348,160 singletons. Additionally, we identified and experimentally validated differentially expressed gene (DEG) transcripts. Finally, using the numerous transcript sequences, we analyze the AS and TE events of crab-eating macaque.

## Results and discussion

### GS FLX sequencing and gene annotation

**Figure 1:**
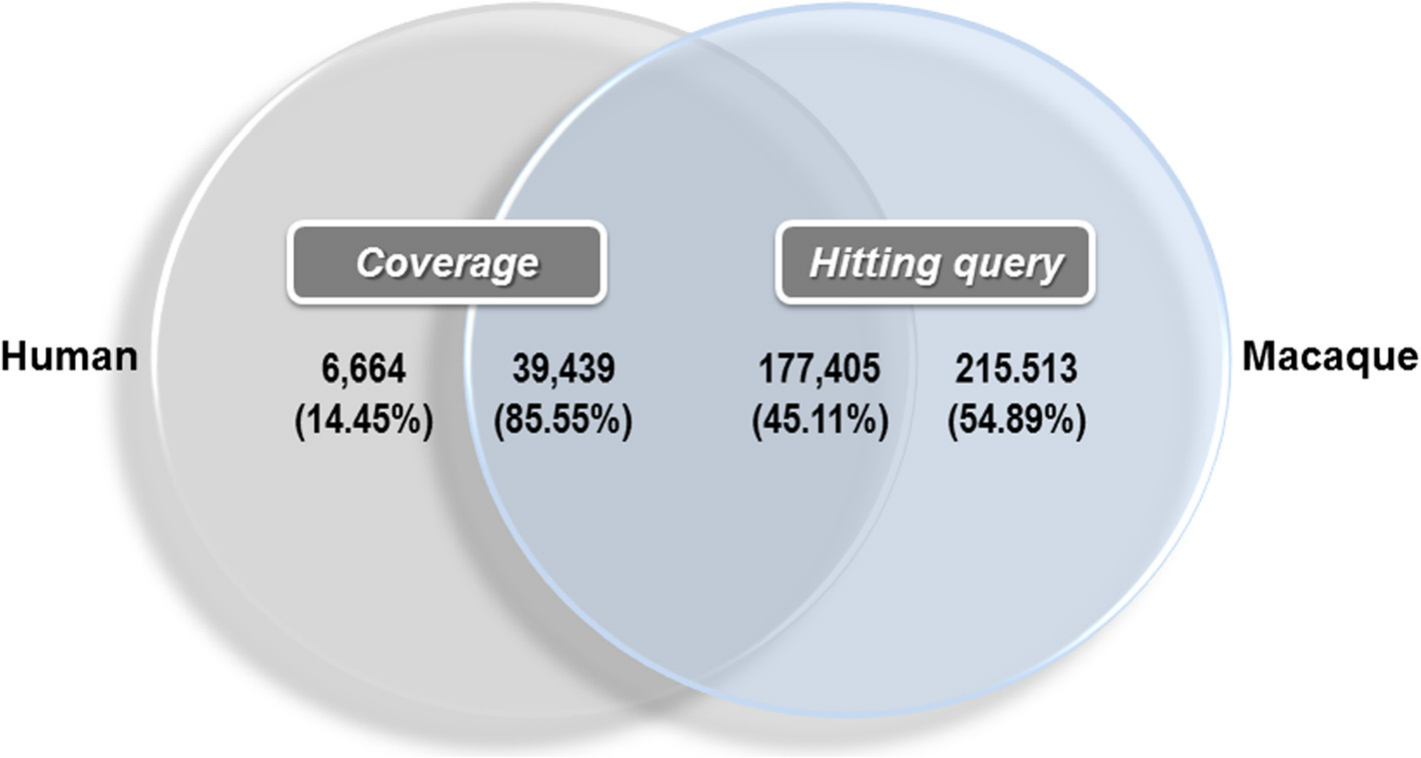
**Comparative analysis of crab-eating macaque transcriptome sequences with human reference genes.** Human reference gene coverage was calculated using the BLASTX program. A total of 177,405 crab-eating macaque transcripts (45.11%) were matched to 39,439 human reference RNA sequences (85.55%).

Among the different next generation sequencing methods, we selected the GS FLX sequencing platform. Although this platform demanded the high cost for sequencing, longer read length of output sequences are more adequate for the de novo assembly for crab-eating macaque genes [61,62]. A total of 4,058,656 raw reads were obtained from the 16 different tissue libraries, with a mean sequenced size length of 355 bp ( Additional file 1: Table S1, Additional file 1: Table S2, Additional file 1: Table S3). For rapid assembly and exact gene annotation, all raw reads were divided into 2 groups, clustered reads and unclustered reads, by the clustering method of the BLASTN program with human reference RNA, generating 3,240,337 reads clustered with human reference RNA, and 818,319 unclustered reads ( Additional file 2: Figure S1). Each group was analyzed by *GS de novo* Assembler v.2.5.3 (Newbler, 454 Life Science). In the clustered group, 38,750 assembled contigs, 31,786 isotigs, and 24,884 isogroups and 99,283 unassembled singletons were generated. However, 132,121 reads were discarded due to excessively short, chimeric, or repetitive sequences. For the clustered isotigs, half of the sequences were larger than 900 bp, and more than 2,400 were longer than 3,000 bp ( Additional file 2: Figure S2). Total annotated sequences covered ~86% (39,439 genes) of the human reference genes (Figure 1; Additional file 1: Tables S4 S5). By contrast, 55% of the sequences (5,915 isotigs and 209,598 singletons) did not match any of the human reference genes ( Additional file 2: Figure S1). Although more detailed experimental validations must be performed, these sequences (5,915 isotigs and 209,598 singletons) may be macaque-specific genes that define differences between humans and crab-eating macaques.

### Application for OMIM database and KEGG pathway database

We then applied our results to the OMIM database (http://www.ncbi.nlm.nih.gov/omim/), which provides information on disorder-related genes that have been functionally well-characterized, and the KEGG pathway database (http://www.genome.jp/kegg/pathway.html), a representative molecular pathway database specifically for disease-related pathways. In the OMIM database, we collected all of the available gene sets for calculation of coverage. Of the 2,579 disorder-related genes in the OMIM database (Additional file 1: Table S6), 1,935 genes (75.02%) were covered by our results (Additional file 1: Table S7), indicating that the gene information from our sequencing could lead to an enhanced understanding of the genetic responses to specific experimental conditions in disease-related research on the crab-eating macaque.

MPTP treatment of the crab-eating macaque is one of the most well established models of Parkinson’s disease [39]. Therefore, we applied our results to the investigation of Parkinson’s disease (map05012) in the KEGG pathway. In general, first step of disease mechanism research is the identification of full-length gene sequences, specifically coding sequences (CDS), using cDNA library or RACE experiments for the investigation of a specific disease. Then other following steps of in vitro or in vivo experiments are applied for the characterization of specific disease. Therefore, the identification of intact CDS in genes was our primary goal. In the KEGG pathway database, 129 Parkinson’s disease genes were registered. We manually tested the existence of open-reading frame sequences and compared the existence of full-length CDS with our sequencing data (data not shown). Our results indicated that total 115 genes (89%) harbor the intact full-length CDS (101 genes) or truncated CDS or UTR sequences (14 genes). These high rate of identification of intact full-length sequences are coincided the property of GS FLX sequencing platform (long-read sequencing) [61,62]. Although, we did not validated the other disease-related genes in OMIM database, our results can clearly reduce the cost and experimental efforts for the identification of specific disorder-related genes for biomedical research.

### Differentially expressed gene analysis and experimental validation

**Figure 2:**
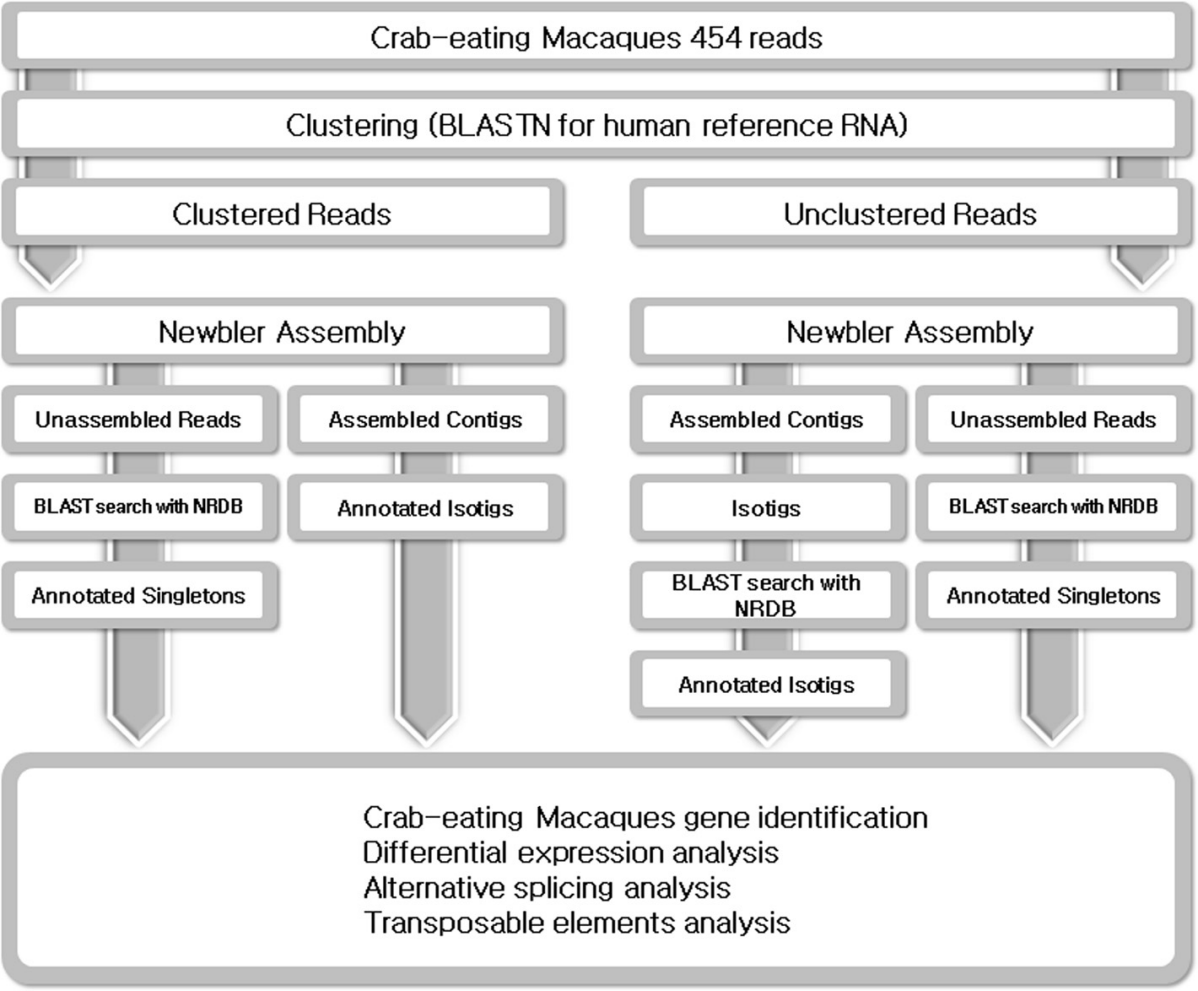
Flow chart for data analysis of the crab-eating macaque.

More than 4 million reads harboring tissue information were used in the assembly steps (Figure 2). Therefore, it was possible to use tissue information to identify differentially expressed genes (DEGs) candidates. Strict filtering conditions were applied for the identification of DEG candidates (more than 100 reads and the use of contigs exclusively expressed in specific tissues). In total, 175 genes were identified as DEG candidates (Additional file 1: Tables S8–S20). Testis (45 genes) and liver (42 genes) showed the largest number of DEG candidates (Table 1). By contrast, the ovary, spleen, cerebrum, and cerebellum did not harbor tissue-specific transcripts. However, when we pooled the cerebrum and cerebellum tissue as brain tissue, one gene, CBLN1 was identified as a DEG candidates.

**Table 1:** Identification and validation of tissue-specific transcripts.

**Tissue**	**DEG candidates**	**Selected DEGs for experimental validation**	Gene Name^*^
Cecum	4	3	*SLC12A2*^1^, *CA1*^2^, *CLCA4*^3^
Cerebellum	1	1	*CBLN1*^4^
Cerebrum			
Heart	3	2	*MYBPC3*^5^, *LDBD3*^6^
Kidney	11	10	*UMOD_T2*^7^, *UMOD_T1*^8^, *TINAG*^9^, *SLC34A1*^10^, *SLC22A6*^11^, *SLC22A12*^12^, *LRP2*^13^, *CDH16*^14^, *C12orf59*^15^, *A2LD1*^16^
Liver	42	10	*CYP2B6*^17^, *C9*^18^, *F9*^19^, *TAT*^20^, *F13B*^21^, *CRP*^22^, *C8B*^23^, *FGG*^24^, *GC*^25^, *MBL2*^26^
Lung	5	4	*SFTPD*^27^, *SFTPB*^28^, *SFTPA1*^29^, *SFTPC*^30^
Ovary^†^	0	0	
Pancreas	22	11	*CELA2A*^31^*, CPB1*^32^*, PRSS3*^33^*, CEL*^34^*, INS*^35^*, CTRB2*^36^*, CELA1*^37^*, CLPS*^38^*, PRSS2*^39^*, CELA3A*^40^*, CPA2*^41^
Prostate	3	2	*SEMG2*^42^*, MSMB*^43^
Salivary gland	19	11	*CA6*^44^*, C4orf40*^45^*, MUC7*^46^*, CST2*^47^*, CST5*^48^*, AMY2A*^49^*, PRB1*^50^*, CST4*^51^*, PRB3*^52^*, STATH*^53^*, HTN1*^54^
Skeletal muscle	11	8	*MYH4*^55^*, AMPD1*^56^*, TPM3*^57^*, ATP2A1*^58^*, MYOT*^59^*, MYBPC1*^60^*, MYL1*^61^*, TNNI2*^62^
Small intestine	2	2	*FABP2*^63^*, DEFA6*^64^
Spleen	0	0	
Stomach	7	5	*CHIA*^65^*, LIPF*^66^*, GKN2*^67^*, GKN1*^68^*, PGA5*^69^
Testis	45	12	*ADAM32*^70^*, SHCBP1L*^71^*, ACRBP*^72^*, CABS1*^73^*, CRISP2*^74^*, TCP11*^75^*, ALLC*^76^*, TUBA3D*^77^*, ANKRD7*^78^*, LDHC*^79^*, CMTM2*^80^*, FUNDC2*^81^

**Figure 3:**
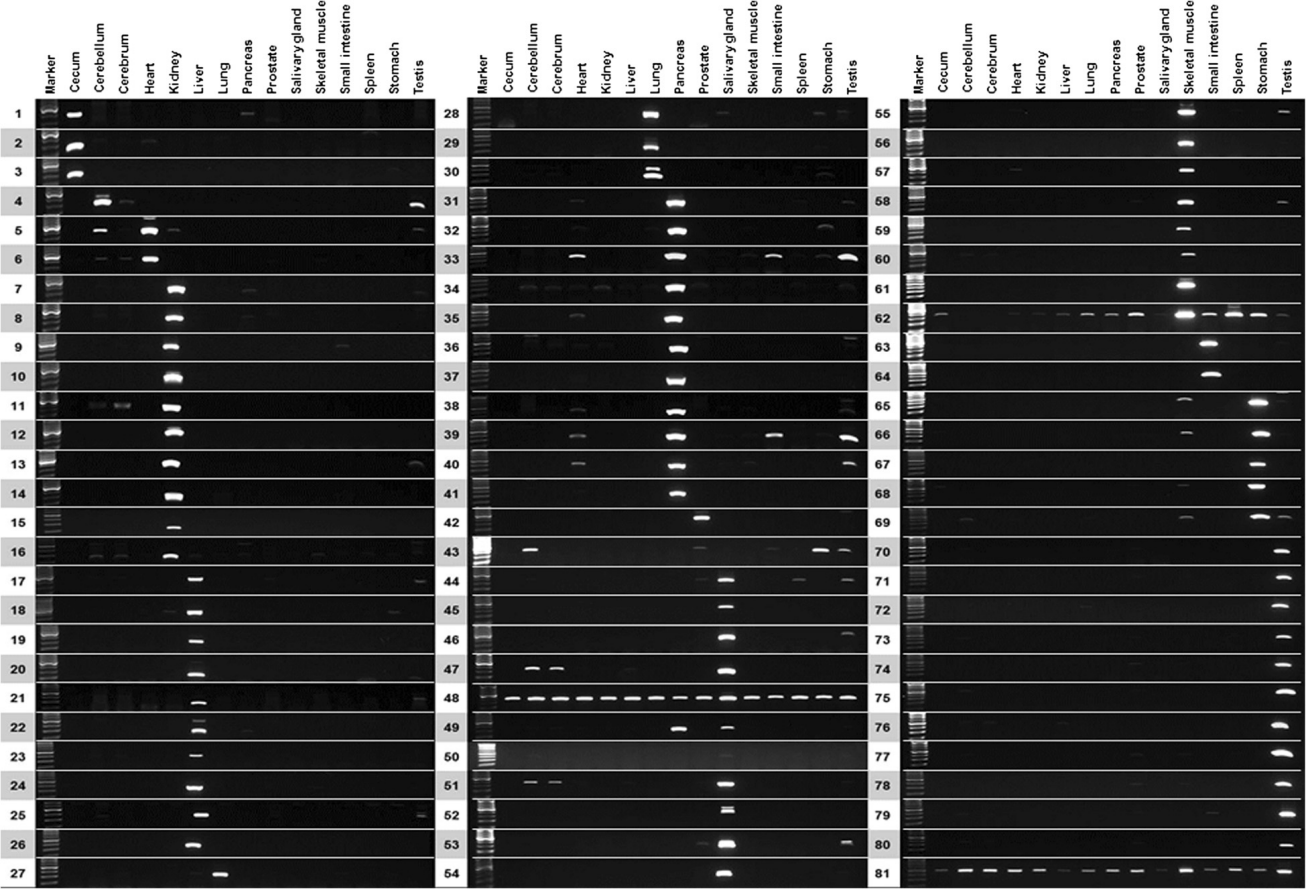
**Experimental validation of DEG candidates.** RT-PCR amplification was conducted with crab-eating macaque tissue samples. To confirm the expected amplification, sequencing was performed.

*The superscript numbers (1–81) correspond to the validated gene numbers in Figure 3.

^†^ Ovary samples were not used for experimental validation for the experimental efficiency.

Identified DEG candidates were subdivided into 3 groups: functionally well-characterized genes in specific tissues, functionally well characterized genes with tissue relatedness not investigated, and functionally not characterized genes with tissue relatedness not investigated. For example, among the 45 testis DEGs, genes including *COX6B2DPY19L2IZUMO4PRM2TSSK6*, and *H1FNT* have been previously investigated as testis-specific transcripts or spermatogenesis-related genes (http://www.ncbi.nlm.nih.gov/gene/). Other genes such as *C6orf225C20orf107FUNDC2*, and *LELP1* have not been functionally investigated in any other tissues in previous research, while the *CETN1* gene has a specific function in centrosome positioning and segregation [63] but has not been investigated with respect to tissue relatedness. Therefore, these DEGs could be utilized as major target genes for tissue specific transcripts for tissue specific function and novel gene identification in specific tissues. For the experimental validation of DEG candidates, 81 genes were randomly selected and experimentally confirmed by RT-PCR amplification and sequencing procedures (Table 1; Figure 3). Remarkably, more than 95% of the genes were validated as real DEGs with distinct expression in expected tissues. These results support the reliability of our sequencing and emphasize the importance of tissue sample preparation when conducting high-throughput sequencing.

### Alternative splicing (AS) analysis

A total of 6,931 manually corrected AS events were identified in the 24,884 clustered isogroups (Additional file 1: Table S21). Total 4314 isogroup harbored the more than one alternatively spliced transcripts. The average number of AS events was 1.60, and the highest number observed was 63 AS events in the *AKR1B10* gene (Additional file 1: Table S22). Intriguingly, the human *AKR1B10* gene shows only one reference mRNA sequence, while the EBI database of Alternative Splicing and Transcript Diversity 1.1 indicated only 5 alternative transcripts for this gene in humans (http://www.ebi.ac.uk/asd/index.html). A careful analysis indicated that AS events occurred more frequently in the 5′ and 3′ regions (2,270 and 2,313, respectively) than the internal regions (1,727) (Additional file 1: Table S22). Further, 274 AS events (10.4%) were TE related. As a result, ~17% of the crab-eating macaque isogroups were shown to have alternatively spliced transcripts. This lower rate of AS events in the crab-eating macaque may be explained by 2 alternative interpretations. One is the shortage of total amount of transcript sequences. In the case of human studies, earlier researches indicated that approximately 40%–70% of genes have alternative transcripts. However, advanced high-throughput sequencing and bioinformatic tools have shown that 92%–95% of human genes undergo AS [50,51,64-66]. In addition, different human tissues show different AS patterns because of tissue-specific alternative promoters and polyadenylation [50-52]. Therefore, larger amount of transcript sequences and more diverse tissues or cell types could enhance the AS information. Another is explained by simple lineage specific characters. Because, we already observed the differential alternative splicing between human and chimpanzees [63,67]. And, as indicated in the genome project of chimpanzee and orangutan, different amplification rate and lineage specific of transposable elements could cause the different TE-derive alternative splicing [68,69].

### Transposable element (TE) analysis

Recent growing genomic evidence has indicated that TEs are a valuable genetic resource for transcriptome and proteome diversity [70-73]. Exonization events are one of the AS mechanisms that can occur as a result of TEs, including human endogenous retroviruses (HERVs), short interspersed elements (SINEs), and long interspersed elements (LINEs). *Alu* (a primate-specific SINE) and LINEs have potential 5′ and 3′ splicing sites for exonization events. Moreover, HERVs and LINEs harbor internal promoters that can control the tissue-specific expression of a gene [59].

**Figure 4:**
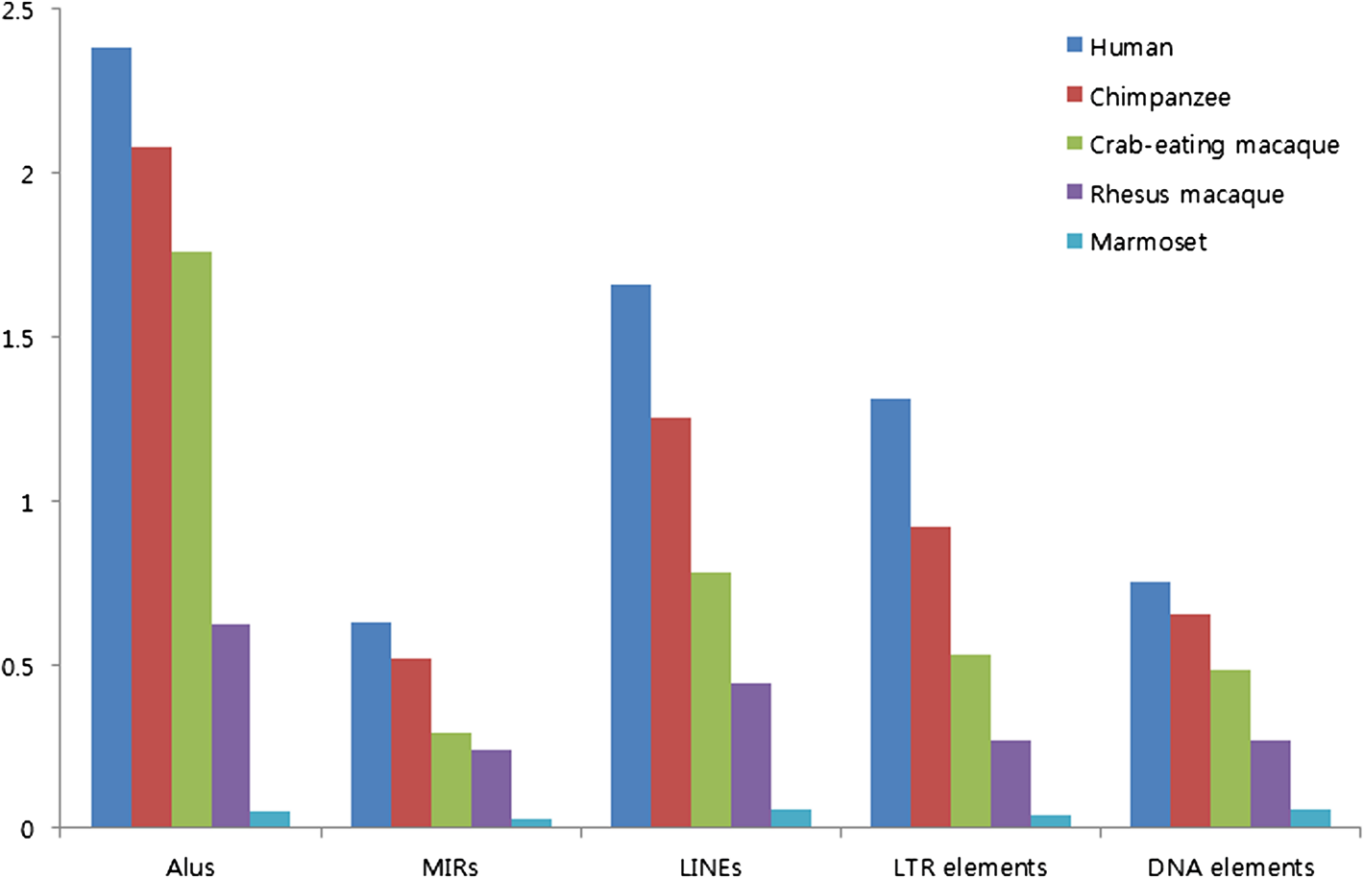
**Comparative analysis of transposable element exonization events in primates.** Human, chimpanzee, crab-eating macaque, rhesus macaque, and marmoset monkey gene information were used for our analysis.

Among the different TEs, *Alu* is the most frequently exonized element. However, in our comparative analysis with human, slight differences in the patterns of *Alu* exonization were observed. *Alu* elements underwent an exonization event in 2.38% of human genes and in 1.76% of crab-eating macaque genes. Therefore, we extended our analysis to all TEs in human, chimpanzee, crab-eating macaque, rhesus macaque, and marmoset monkey for the comparative analysis of primates. Intriguingly, this extended study indicated a increase pattern in TE composition over primate evolution and different TE-exonization events between rhesus macaque and cran-eating macaque (Figure 4). Although primate gene information was not sufficient to conclude from our results that amplified TE composition is a human-specific event, our results do indicate that TE exonization events were amplified over primate evolution and notably in humans. These types of amplified TE exonization events in humans could enhance the transcriptome and proteome diversity with fixed genome sequences in comparison with non-human primates. However, we also explained the results of Figure 4 as decrease pattern in TE composition. Because the probability is very low, recent studies newly raised the *Alu* recombination-mediated deletion (ARMD) and L1 recombination-associated deletions (LRMD) mechanisms which could remove the internal sequences by homologous recombination of “*Alu*” or “LINE” elements [74,75]. In the case of rhesus macaque and marmoset, the results of low-level TE-exonization rate in comparison with other species are seems to be occurred by the lack of transcript sequences (http://www.ncbi.nlm.nih.gov/Taxonomy/Browser/). Because most of reference mRNA sequences are identified by computational screening without the intensive support of numerous EST or cDNA sequences.

### Broad range of utility of crab-eating macaque gene information

The results of our study have implications for various fields of research. First, the massive number of transcriptome sequences (approximately 4 million sequences in 16 tissues) could be used as a draft of the crab-eating macaque gene sequences. In addition, the modified and combined gene information could be used for the production of DNA probe sequences for microarray analysis. Specifically, the company Agilent provides a customized probe design service using industrial-scale inkjet technology (http://www.genomics.agilent.com/). Therefore, crab-eating macaque microarray chips could be designed for specific experiments and more rapid and accurate gene expression profiling is possible in a single experiment. For example, to investigate specific drugs for Parkinson’s disease, customized microarray chips harboring the 129 Parkinson’s disease-related crab-eating macaque genes from the KEGG pathway database could be prepared.

Second, crab-eating macaque gene information coupled with gene information from the rhesus macaque could be used to resolve the mystery of speciation events between closely related species. The average genetic divergence between crab-eating macaque and rhesus macaque is 0.4%–0.5%, and their evolutionary relationship is closer than that between human and chimpanzee [14]. Therefore, large-scale transcript sequences could help to trace the evolutionary root of the speciation event. Third, gene sequencing of the crab-eating macaque could accelerate the completion of a genome project for this primate. Recently draft genome sequences also available (http://www.ebi.ac.uk/asd/index.html). Hence, reanalysis and diverse application could be possible for the analysis of genome and transcripme in crab-eating macaque. Fourth, the 175 DEGs, including the 81 experimentally validated DEGs, represent candidate genes with tissue-specific functions. Specifically, two of gene groups of functionally well characterized genes with tissue relatedness not investigated, and functionally not characterized genes with tissue relatedness not investigated, could be a valuable sources for tissue specific functional study and novel function analysis in specific tissue, respectively. Fifth, the AS and TE exonization analysis could be used for comparative analysis of crab-eating macaque with other species. Although, the data set are not sufficient for other application, our results are to be used as basic information to understand the transcriptome of crab-eating macaque. Finally, our open data base are very useful for numerous researchers who are interested in the gene information of crab-eating macaque, specifically unskilled researchers in genomics and bioinformatics technique.

## Conclusions

We sequenced the transcriptome of 16 different tissues from M. fascicularis for the biomedical usage. We found that ~86% of human genes are represented in the ones sequenced in this study. Therefore our results of gene information could be used for understanding the biological response of the crab-eating macaque for safety and efficacy testing. Additionally, 175 tissue-specific genes were identified, with 81 of them experimentally validated. We identified and analyzed 4,314 alternative splicing (AS) events and positive selected genes. Intriguingly, 10.4% of the AS events were associated with transposable element (TE) insertions. And human-specific amplified trends of TE exonization event are also revealed during the primate evolution. Our research is the first large-scale transcriptome sequencing and gene analyses. Therefore, this result could be valuable genetic resources for biomedical research and improve our understanding of primate evolution.

## Methods

### Specific pathogen free (SPF) crab-eating macaques

Adult male (5 years old) and female (6 years old) crab-eating macaques (Macaca fascicularis) weighing between 4 kg and 7 kg were used. Their origin is vietnam. All animals were provided by the National Primate Research Center (NPRC) of Korea. In our experiments, specific pathogen free (SPF) animals were used. All animals underwent a complete physical, viral, bacterial, and parasite examination. On physical examination, SPF animals were examined for criteria, including coat condition, appearance, weight, sex, and date of birth. Enzyme immunoassay was performed to detect viruses such as BV; STLV-1 and −2; SIV; SRV-1, -2, and −5; and SVV. In addition, tests were performed to detect *Mycobacterium tuberculosis* (TB), *Shigella* spp., *Salmonella* spp., and *Yersinia* spp. For the TB skin test, all animals were tested by an intradermal injection in the eyelid, and the remaining bacterial examination items were checked by fecal culture tests. In our SPF animals, all items in the above tests were negative.

### Sample preparation for GS FLX sequencing and gene annotation

The most important issue for transcriptome sequencing is the preparation of fresh and healthy tissue samples. Therefore, specific pathogen free (SPF) one male and one female adult crab-eating macaques were selected. Additionally, perfusion with diethylpyrocarbonate (DEPC)-treated phosphate buffered saline (PBS) was conducted via the common carotid artery with RNase inhibitors to inhibit blood contamination and promote recovery of intact RNA molecules from the tissue samples. Sixteen tissue samples were collected from one male and one female crab-eating monkeys (1. Cecum, 2. Cerebellum, 3. Heart, 4. Kidney, 5. Liver, 6. Lung, 7. Ovary, 8. Pancreas, 9. Prostate, 10. Salivary gland, 11. Skeletal muscle, 12. Small intestine, 13. Spleen, 14. Stomach, 15. Testis, and 16. Cerebrum).

### Ethics statement

All animal procedures and study design were conducted in accordance with the Guidelines of the Institutional Animal Care and Use Committee (KRIBB-AEC-11010) in Korea Research Institute of Bioscience and Biotechnology (KRIBB).

### RNA isolation and mRNA subtraction

Total RNA was extracted from 16 different crab-eating monkey tissues using the Trizol reagent (Invitrogen), and total RNAs were validated by RNA electrophoresis in agarose gels containing formaldehyde. Two distinct ribosomal RNA bands (28 S and 18 S) were confirmed. Pure mRNA was subtracted using the PolyA Tract mRNA isolation system (Promega).

### cDNA synthesis and poly(A) tail removal

First strand cDNA synthesis was conducted using the RevertAid H Minus First Strand cDNA Synthesis Kit (Fermentas) using oligo(dT) primers optimized for the 454 sequencing procedures (5′- GAGCTAGTTCTGGAG(T)_16_VN-3′). Second strand cDNA was synthesized by DNA pol I and RNase H (Fermentas), and the poly(A) tail was removed using a specific enzyme (Gsul).

### Library preparation for GS FLX sequencing

The first step of library preparation involves the fragmentation of the high molecular weight DNA sample into smaller molecular species appropriate for sequencing using GS FLX Titanium chemistry. This fragmentation is performed by nebulization, which shears double-stranded DNA into fragments ranging from about 400 to 1000 base pairs. This population of smaller-sized DNA species, generated from a single DNA sample, is referred to as a “library.” Approximately 3–5 μg cDNA was used to generate the DNA library for Genome Sequencer FLX Titanium (Roche, Mannheim, GE). The fragment ends were polished (blunted), and 2 short adapters were ligated onto both ends. The adapters provide priming sequences for both amplification and sequencing of the sample library fragments, as well as the “sequencing key”, a short sequence of 4 nucleotides used by the system software for base calling and, following repair of any nicks in the double-stranded library, release of the unbound strand of each fragment (with 5′-Adaptor A). Finally the quality of the library of single-stranded template DNA fragments (sst DNA library) was assessed using a 2100 BioAnalyzer (Agilent, Waldbronn, GE), and the library was quantified, including a functional quantification to determine the optimal amount of the library to use as input for emulsion-based clonal amplification.

### Emulsion PCR

Single “effective” copies of template species from the DNA library to be sequenced were hybridized to DNA Capture Beads. The immobilized library was then resuspended in the amplification solution, and the mixture was emulsified, followed by PCR amplification. After amplification, the DNA-carrying beads were recovered from the emulsion and enriched. The second strands of the amplification products were melted away as part of the enrichment process, leaving the amplified single-stranded DNA library bound to the beads. The sequencing primer was then annealed to the immobilized amplified DNA templates.

### Sequencing

After amplification, the DNA-carrying beads were set into the wells of five and a half PicoTiterPlate device (PTP) such that each well contained a single DNA bead. The loaded PTP was then inserted into the Genome Sequencer FLX instrument, and sequencing reagents were sequentially flowed over the plate. Information from all the wells of the PTP is captured simultaneously by a camera and can be processed in real time by the onboard computer. The sequencing procedure was conducted on a Genome Sequencer FLX Titanium instrument (Roche, Mannheim, GE) at Macrogen in Korea.

### Sequence assembly and gene annotation

A total of 4,058,656 raw reads obtained from the 16 libraries were used for our analysis. For rapid assembly and exact gene annotation, all raw reads into were divided into 2 groups, clustered reads and unclustered reads, by the clustering method of the BLASTN program with human reference RNA ( Additional file 2: Figure S2). This method generated 3,240,337 reads clustered with human reference RNA and 818,319 unclustered reads. Each group was analyzed by *GS de novo* Assembler v.2.5.3 (Newbler, 454 Life Science). The clustered group generated 38,750 assembled contigs, 31,786 isotigs, and 24,884 isogroups and 99,283 unassembled singletons. However, 132,121 reads were discarded due to short, chimeric, or repetitive sequences. The unclustered group generated 16,108 assembled contigs 12,672 isotigs, and 10,640 isogroups and 248,877 unassembled singletons. In addition, 57,613 reads were also discarded.

Two different gene annotation strategies were conducted in the clustered and unclustered groups. In the clustered group, initial gene information obtained by clustering with human reference RNA was used for the gene annotation. However, in the case of the unclustered group and unassembled singleton sequences, the BLASTX program was used with the nr70 database. The CD-HIT program (http://www.bioinformatics.org/cd-hit/) was used to build the nr70 database. If gene annotation was conducted, Gene Ontology (GO) searching (http://www.geneontology.org/) and Kyoto Encyclopedia of Genes and Genomes (KEGG) analysis (http://www.genome.jp/kegg/) were performed.

### KEGG pathway analyses

By overlaying expression data onto biological pathways, established and novel relationships among genes can be explored. These pathways give key information about the functional and metabolic organization of cellular and biological systems within organisms. Therefore, putative crab-eating macaque genes incorporate KEGG pathway information. The pathway analysis pipeline extracts EC numbers from the descriptions of UniProt results, and these EC numbers are mapped with KEGG pathway information.

### Coverage calculation

Using the annotated gene information, our sequences were compared with human unigene and reference sequences. Our sequences were analyzed using the BLASTN program with an expectation value of 1e^-20^. If one match occurred between human and crab-eating macaque sequences, the one match was interpreted as a covered result. Additionally, Online Mendelian Inheritance in Man (OMIM) gene sets were applied for disease-related gene research (http://www.ncbi.nlm.nih.gov/omim).

### Differentially expressed gene (DEG) analysis

Sixteen different tissue samples were collected and sequenced. Thus, over 4 million reads harboring different tissue information were available for the DEG analysis. DEG information was extracted by counting the read information. Exclusively tissue-specific contigs (only allowed 100%) that contained a minimum of 100 reads were selected. For the experimental validation, 81 randomly selected DEGs were validated.

### Reverse transcriptase polymerase chain reaction (RT-PCR) amplification and sequencing procedure

Locus-specific primer pairs were used for the RT-PCR amplification of 81 DEGs (Additional file 1: Table S23). If possible, 2 distant exons were used for constructing primer pairs to reduce non-specific PCR bands resulting from genomic contamination. In the validation steps, 15 tissues samples are used for the experimental efficiency (We removed the ovary samples). M-MLV reverse transcriptase with an annealing temperature of 42°C was used for the reverse transcription reaction with an RNase inhibitor (Promega). Control PCR amplification was also performed on pure mRNA samples that were not subjected to reverse transcription, indicating that the prepared mRNA samples did not contain genomic DNA. RT-PCRs were carried out for 30 cycles at specific annealing temperatures. To validate amplified products, RT-PCR products were separated on a 1.5% agarose gel, purified using a gel extraction kit (GeneAll), and cloned into the pGEM-T-easy vector (Promega). The cloned DNA was isolated using a plasmid DNA mini-prep kit (GeneAll). Sequencing was conducted by a commercial sequencing company (Macrogen).

### Transposable element (TE) analysis

The TEs included in the human reference RNAs, chimpanzee reference RNAs, rhesus reference RNAs, marmoset reference RNAs, and clustered assembly contigs were analyzed for comparative TE analysis. The TEs were identified by the RepeatMasker program (http://repeatmasker.genome.washington.edu) with various repeat sequences from the Repbase Update.

### Alternative splicing (AS) analysis

For the AS analysis, the Newbler2.5 assembly result files (54AllContig.fna, 454Isotigs.fna, and 454IsotigsLayout.txt) were modified. Among these result files, the 454IsotigsLayour.txt file demonstrated the relationships between isotigs and contigs in specific isogroups. Therefore, the alternatively spliced isogroup information was collected. Among the AS data, only clustered and annotated isogroups were analyzed for the comparative analysis with humans. However, in the case of crab-eating macaque, detailed phenomena could not be investigated because no crab-eating macaque genome sequences are available. For a detailed analysis, the AS data was analyzed manually. The 5′ and 3′ alternative exon and internal exon units that could occur by exon creation or loss ( Additional file 1: Table S22) and the TE-related AS were counted. In the manual analysis, specific exons harboring a TE in the marginal regions of exons were designated as TE-related AS (Additional file 2: Figure S3).

## Abbreviations

AS = Alternative Splicing; BP = Biological Process; CC = Cellular Component; CDS = Coding Sequences; DEG = Differentially Expressed Gene; DEPC = Diethylpyrocarbonate; GO = Gene Ontology; HERVs = Human Endogenous Retroviruses; KEGG = Kyoto Encyclopedia of Genes and Genomes; LINEs = Long Interspersed Elements; MPTP = 1-methyl-4-phenyl-1,2,3,6-tetrahydropyridine; NIH = National Institutes of Health; NPRC = National Primate Research Center; OMIM = Online Mendelian Inheritance in Man; PBS = Phosphate Buffered Saline; PTP = PicoTiterPlate; RT-PCR = Reverse Transcriptase Polymerase Chain Reaction; SINEs = Short Interspersed Elements; SPF = Specific Pathogen Free; TB = Tuberculosis; TE = Transposable Element.

## Competing interests

Authors declare that they have no competing interests.

## Authors’ contributions

KTC managed the project. JWH analyzed the sequencing data. JWH, YHK and SJP wrote the manuscript. DSK conducted the bioinformatic analysis. KMK, KJJ and SRL conducted the housing and sampling the crab-eating macaques. YHK, SJP, BSS, JSK, BWS, SUK and SHK validated the sequencing data. All authors read and approved the final manuscript.

## Accession numbers and database

The data have been deposited in the DDBJ under accession number DRA000436. The assembled sequences are also freely available from http://203.239.28.13/macaca/.

## Supplementary Material

Additional file 1 **Table S1.** The information of GS FLX sequencing procedure. **Table S2.** The summary of sequencing procedure in 16 different tissues. **Table S3.** The summary of Crab-eating Macaques 454 sequencing. **Table S4.** Coverage calculation of crab-eating macaque through human unigene and human reference gene. **Table S5.** Calculation of hitting query of crab-eating macaque with human unigene and human reference. **Table S6.** The list of Gens used for OMIM analysis. **Table S7.** The list of OMIM genes covered by crab-eating macaque. **Table S8.** The list of DEG candidate in Brain. **Table S9.** The list of DEG candidate in Cecum. **Table S10.** The list of DEG candidate in Heart. **Table S11.** The list of DEG candidate in Kidney. **Table S12.** The list of DEG candidate in Liver. **Table S13.** The list of DEG candidate in Lung. **Table S14.** The list of DEG candidate in Pancreas. **Table S15.** The list of DEG candidate in Prostate. **Table S16.** The list of DEG candidate in Salivary gland. **Table S17.** The list of DEG candidate in Skeletal muscle. **Table S18.** The list of DEG candidate in Small intestine. **Table S19.** The list of DEG candidate in Stomach. **Table S20.** The list of DEG candidate in Testis. **Table S21.** Summary of alternative splicing events in crab-eating macaque. **Table S22.** Manually analyzed results of alternative splicing in crab-eating macaque. **Table S23.** Primer infromation for DEG validation. Click here for file

Additional file 2 **Figure S1.** Flowchart for bioinformatic analysis. **Figure S2.** Length distribution of crab-eating macaque isotigs. For the analysis of length distribution, clustered and unclustered isotigs were analyzed. **Figure S3.** Manual selection method for TE-derived AS events. Click here for file
