# Supplementary figures and images for: Large-scale transcriptome sequencing and gene analyses in the crab-eating macaque (Macaca fascicularis) for biomedical research
Source: BMC Genomics. 2012 May 4;13:163. doi: 10.1186/1471-2164-13-163 (PMC3496626; doi:10.1186/1471-2164-13-163)

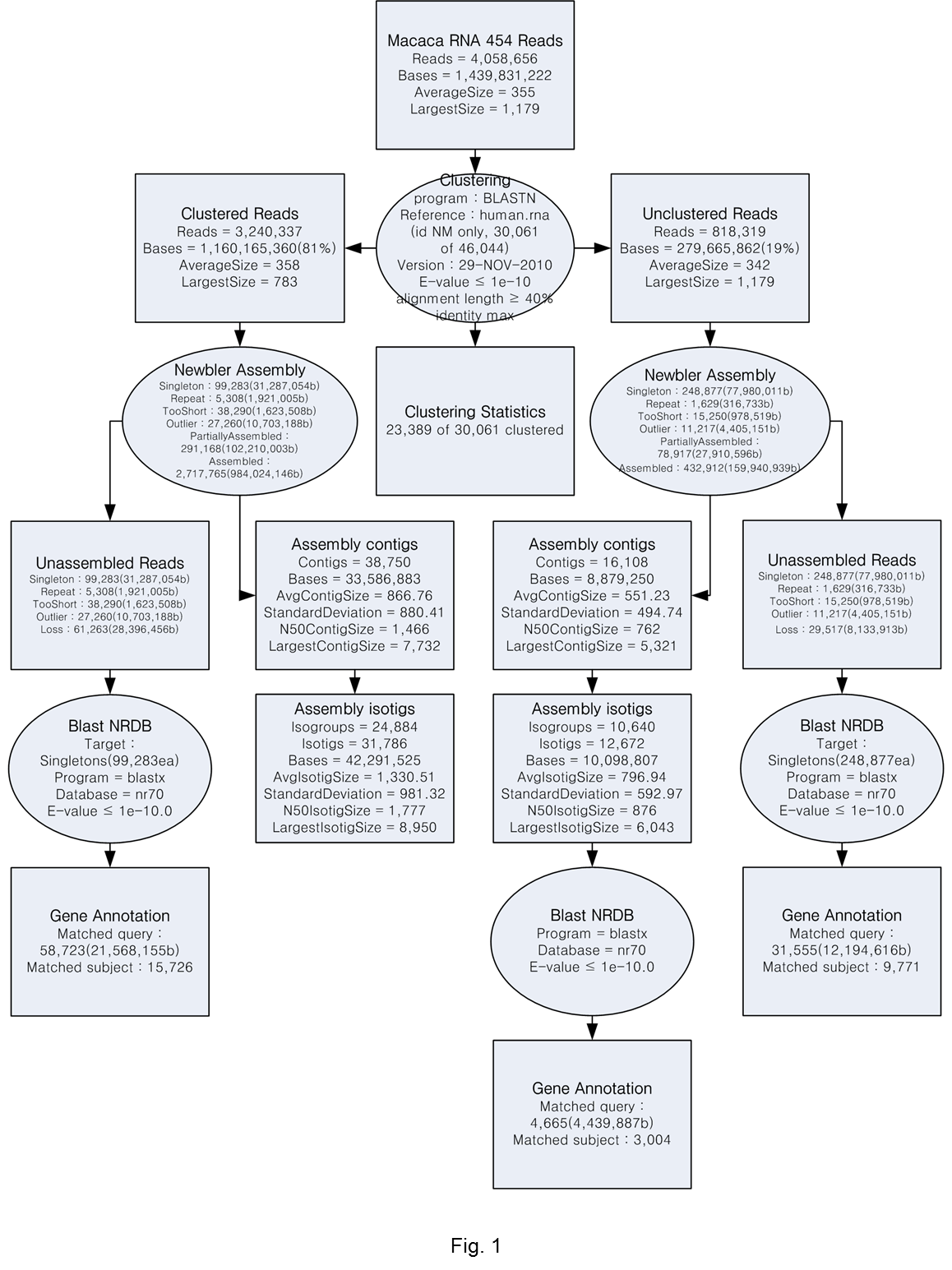

Supplement: Additional file 1 — Table S1. The information of GS FLX sequencing procedure. Table S2. The summary of sequencing procedure in 16 different tissues. Table S3. The summary of Crab-eating Macaques 454 sequencing. Table S4. Coverage calculation of crab-eating macaque through human unigene and human reference gene. Table S5. Calculation of hitting query of crab-eating macaque with human unigene and human reference. Table S6. The list of Gens used for OMIM analysis. Table S7. The list of OMIM genes covered by crab-eating macaque. Table S8. The list of DEG candidate in Brain. Table S9. The list of DEG candidate in Cecum. Table S10. The list of DEG candidate in Heart. Table S11. The list of DEG candidate in Kidney. Table S12. The list of DEG candidate in Liver. Table S13. The list of DEG candidate in Lung. Table S14. The list of DEG candidate in Pancreas. Table S15. The list of DEG candidate in Prostate. Table S16. The list of DEG candidate in Salivary gland. Table S17. The list of DEG candidate in Skeletal muscle. Table S18. The list of DEG candidate in Small intestine. Table S19. The list of DEG candidate in Stomach. Table S20. The list of DEG candidate in Testis. Table S21. Summary of alternative splicing events in crab-eating macaque. Table S22. Manually analyzed results of alternative splicing in crab-eating macaque. Table S23. Primer infromation for DEG validation. [file 1471-2164-13-163-S1.zip › S_Fig. 1.tif]

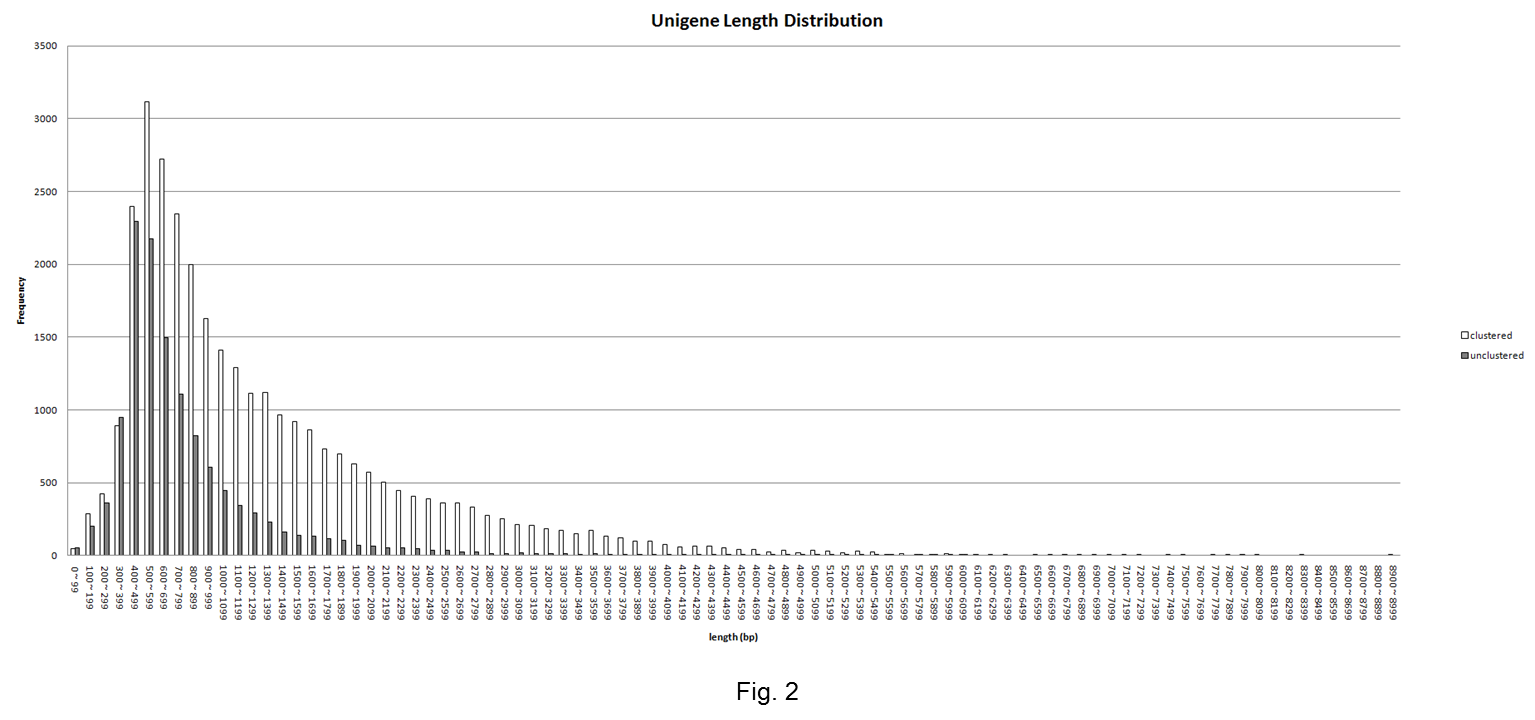

Supplement: Additional file 1 — Table S1. The information of GS FLX sequencing procedure. Table S2. The summary of sequencing procedure in 16 different tissues. Table S3. The summary of Crab-eating Macaques 454 sequencing. Table S4. Coverage calculation of crab-eating macaque through human unigene and human reference gene. Table S5. Calculation of hitting query of crab-eating macaque with human unigene and human reference. Table S6. The list of Gens used for OMIM analysis. Table S7. The list of OMIM genes covered by crab-eating macaque. Table S8. The list of DEG candidate in Brain. Table S9. The list of DEG candidate in Cecum. Table S10. The list of DEG candidate in Heart. Table S11. The list of DEG candidate in Kidney. Table S12. The list of DEG candidate in Liver. Table S13. The list of DEG candidate in Lung. Table S14. The list of DEG candidate in Pancreas. Table S15. The list of DEG candidate in Prostate. Table S16. The list of DEG candidate in Salivary gland. Table S17. The list of DEG candidate in Skeletal muscle. Table S18. The list of DEG candidate in Small intestine. Table S19. The list of DEG candidate in Stomach. Table S20. The list of DEG candidate in Testis. Table S21. Summary of alternative splicing events in crab-eating macaque. Table S22. Manually analyzed results of alternative splicing in crab-eating macaque. Table S23. Primer infromation for DEG validation. [file 1471-2164-13-163-S1.zip › S_Fig. 2.tif]

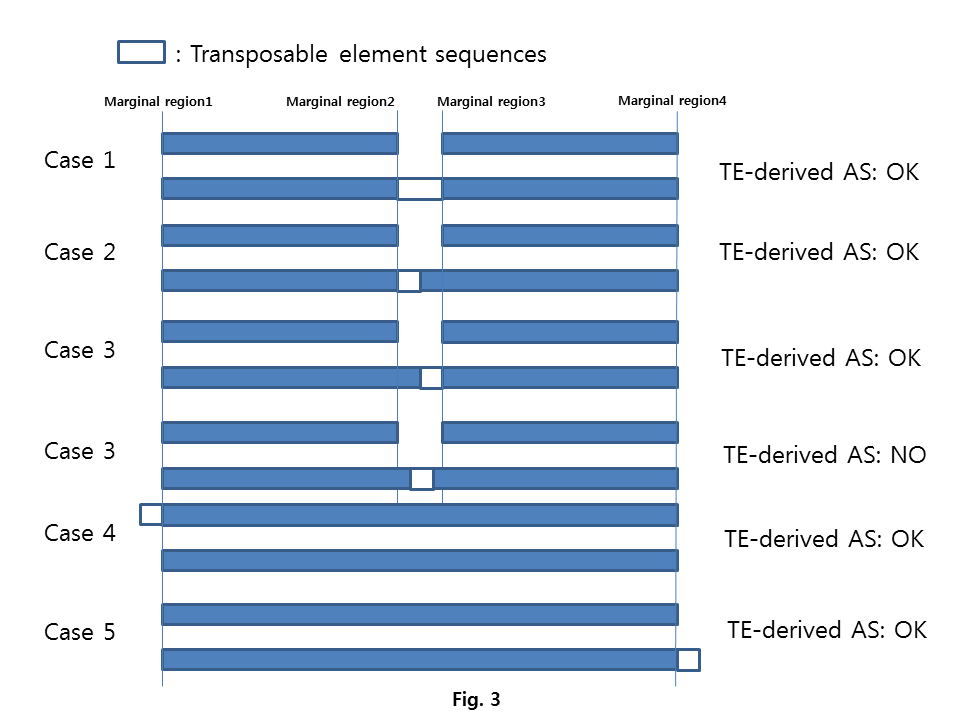

Supplement: Additional file 1 — Table S1. The information of GS FLX sequencing procedure. Table S2. The summary of sequencing procedure in 16 different tissues. Table S3. The summary of Crab-eating Macaques 454 sequencing. Table S4. Coverage calculation of crab-eating macaque through human unigene and human reference gene. Table S5. Calculation of hitting query of crab-eating macaque with human unigene and human reference. Table S6. The list of Gens used for OMIM analysis. Table S7. The list of OMIM genes covered by crab-eating macaque. Table S8. The list of DEG candidate in Brain. Table S9. The list of DEG candidate in Cecum. Table S10. The list of DEG candidate in Heart. Table S11. The list of DEG candidate in Kidney. Table S12. The list of DEG candidate in Liver. Table S13. The list of DEG candidate in Lung. Table S14. The list of DEG candidate in Pancreas. Table S15. The list of DEG candidate in Prostate. Table S16. The list of DEG candidate in Salivary gland. Table S17. The list of DEG candidate in Skeletal muscle. Table S18. The list of DEG candidate in Small intestine. Table S19. The list of DEG candidate in Stomach. Table S20. The list of DEG candidate in Testis. Table S21. Summary of alternative splicing events in crab-eating macaque. Table S22. Manually analyzed results of alternative splicing in crab-eating macaque. Table S23. Primer infromation for DEG validation. [file 1471-2164-13-163-S1.zip › S_Fig. 3.tif]
